# Understanding the Relationship between Situational Strength and Burnout: A Multi-Sample Analysis

**DOI:** 10.3390/ijerph18010162

**Published:** 2020-12-28

**Authors:** José García-Arroyo, Isabel Cárdenas Moncayo, Antonio Ramón Gómez García, Amparo Osca Segovia

**Affiliations:** 1 Faculty of Psychology, National Distance Education University (UNED), 28015 Madrid, Spain; icardenas22@alumno.uned.es (I.C.M.); aosca@psi.uned.es (A.O.S.); 2 Esai Business School, Universidad Espíritu Santo, Samborondón 091650, Ecuador; agomezg@uees.edu.ec

**Keywords:** situational strength, burnout, clarity, consistency, constraints, consequences

## Abstract

Many studies have examined the effect of situational strength (clarity, consistency, constraints, and consequences) on organisational behaviour, but little has been investigated about its health effects. This study aimed to analyse the relationship between situational strength and burnout. Specifically, we examined whether situational strength characteristics may be associated with burnout, whether these characteristics are risk (or protective) factors for burnout, and whether a strong situation is related to higher levels of burnout. Examining three samples from different occupations, it was found that clarity and consistency are negatively associated with burnout, being protective factors, while constraints are positively associated with burnout, being risk factors. These results are consistent across the samples. In addition to the direct effects, interaction effects between clarity and consistency in the office employee’s sample (two-way interaction), between constraints and consequences in the samples of office employees and teachers (two-way interaction), and among clarity, consistency, and constraints in the salespeople’s sample (three-way interaction) were also significant, explaining from 20% to 33% of the variance of burnout. We concluded that situational strength is associated not only with behaviour but also with health. The theoretical and practical implications of these findings are discussed.

## 1. Introduction

Situational strength is defined as the implicit or explicit cues provided by entities external to the individual regarding the appropriateness of certain forms of behaviour [1]. The most used operationalisation of situational strength so far distinguishes four main characteristics (facets), namely, clarity, consistency, constraints, and consequences [2]. These characteristics of a situation can influence (enhance or restrict) the behaviour of a person in a given setting. Situational strength can significantly impact people’s behaviours and even have a greater effect than personality characteristics [2]. When the environmental characteristics determine how an individual has to behave, the situation is strong. On the contrary, if the environmental characteristics allow the subject freedom to decide and act, the situation is weak.

Many studies have examined the effect of situational strength on organisational behaviour [3,4,5], but little has been investigated about its health effects. For example, situations with high situational strength can favour compliance with safety regulations and foster healthy behaviours at work (behavioural outcome), especially in higher-risk jobs, nevertheless they can also increase stress and deterioration of psychological health and well-being of the employee (health outcome) [1,6]. To contribute to a better understanding on health effects, this study aimed to analyse the relationship between situational strength and burnout. Specifically, we examined whether situational strength characteristics may be associated with burnout, whether these characteristics are risk (or protective) factors for burnout, and whether a strong situation is related to higher levels of burnout. Additionally, knowing that the strength of a situation is related to occupation [5,7], and to increase the robustness of the study results, we analysed data from three samples of different occupations.

### 1.1. Situational Strength at Work

Situational strength at work is understood as environmental pressure on the individual in the workplace settings that will influence his/her behaviour in an important way [1]. The meta-analysis by Meyer, Dalal, and Bonaccio [5] examined in 114 studies the different forms of empirically operationalising environmental characteristics and grouped them into two broad categories. These categories are “constraints” and “consequences” and represent two logically consistent dimensions of how situational strength can affect behaviour. Subsequently, after analysing the historical evolution of the concept of situational strength, Meyer et al. [1] included two more factors, clarity and consistency, which theoretically completed the construct of situational strength. Later, this four-factor structure containing clarity, consistency, constraints, and consequences, was operationalised through the Situational Strength at Work Scale [2]. Clarity is defined as the extent to which directions related to job responsibilities and requirements are available and easy to understand. The greater the clarity of information about the expected behaviours of employees, the smaller the differences in the behaviours of those who perform them and, therefore, the more predictable the behaviour is. Consistency refers to the degree to which the indications related to job requirements are compatible with each other and with other indications, that is, to what extent the different sources of information offer consistent information or not on the expected behaviours of employees. The higher the consistency of indications, the greater the uniformity of behaviours. Constraints consist of the degree to which individual freedom to decide or act is limited by forces beyond his/her control. Constraints limit the behaviour of individuals as to what actions to perform, or when and how to perform them. Finally, consequences refer to the degree to which the actions or decisions have important positive or negative implications for other people, for the organisation, or for other situations. This factor influences behaviour since people tend to increase the possibility of positive results and to avoid or minimise negative ones. These four factors are part of the same construct, but each one provides different information so that the strength of each particular situation is a function of the conjoint effect of the four. However, it is not yet clear how the four factors are related and combined [1].

### 1.2. Situational Strength and Burnout

Burnout is considered a highly prevalent globalised health issue [8,9]. It is a syndrome that consists of a response to prolonged exposure to chronic work environment stressors [10] that negatively affect the physical and psychological health of workers as well as their performance. For example, it is associated with physical [11] and mental disorders [12], low morale [13], intention to quit [14], poor performance [15], and work–family conflict [16], among others.

The literature on situational strength and burnout is very scarce. In a search carried out in the PsycInfo, Medline, Psycodoc, and PsycArticles databases in August 2020, no reference was found with the words “situational strength” AND “burnout” OR “health” in the titles or abstracts. Some references were found by searching separately for each situational strength factor. Specifically, regarding clarity, three references were found that were consistent in pointing out the negative association between clarity and burnout. For example, Blumenthal, Lavender, and Hewson [17] compared role clarity and burnout level in two groups of support workers, finding that the group with the highest level of role clarity has less burnout level. Frögli et al. [18] found that role clarity was negatively related to burnout in nurses during the first professional year, and Vullinghs et al. [19] found that role clarity moderated the effect of passive leadership on followers’ burnout. Concerning consistency, a study [20] reported the negative relationship between self-consistency and burnout in principals, so that the less self-consistent and more inefficient they were, the higher burnout they felt. Related to constraints, a reference found [21] indicated that the constraints of the work environment lead workers to experience burnout. Finally, no references about consequences and burnout were found.

More research has been done on the theoretical concepts opposed to clarity, consistency, and constraints. Specifically, according to Meyer et al. [2], role conflict and role ambiguity conceptually overlap with lack of clarity and lack of consistency respectively, and job control and autonomy would express the opposite concept to constraints. Thus, if the relationship between role conflict, role ambiguity, and burnout is positive, it is expected that the direction of the relationship between clarity, consistency, and burnout will be negative. Similarly, if the relationship between job control and burnout is negative, then the relationship between constraints and burnout will be positive. Empirical evidence supports this rationale. Studies analysing the relationship between role conflict, role ambiguity, and burnout are consistent in pointing out positive associations between these concepts, that is, high levels of role conflict and role ambiguity are related to high levels of burnout [22,23,24,25]. In turn, studies that examine job control and autonomy are consistent in pointing out negative relationships to burnout [26,27,28]. Therefore, we present hypothesis 1:

**Hypothesis** **1.** *Clarity and consistency will associate negatively with burnout, whereas constraints and consequences will associate positively with burnout.*

Additionally, drawing on the job demands–resources theory [29], stressors at work are environmental factors, work characteristics, and work events that usually harm the quality of working life as well as employee health, safety, and well-being, whereas resources are usually protective factors [30]. Individuals experience stressors as risk agents that elicit strain reactions such as burnout [31]. From this perspective, clarity and consistency are organisational resources to increase and facilitate understanding of information about job responsibilities and/or requirements and to uniformly communicate a particular course of action through a variety of channels, whereas constraints and consequences are demands that can limit autonomy, job control, and decision-making capacity, generating stress [2]. Accordingly, we present hypothesis 2:

**Hypothesis** **2.** *Clarity and consistency may be protective health factors whereas constraints and consequences may be risk health factors of burnout.*

In summary, although there is not much research on the relationships between the characteristics of situational strength and burnout, the empirical evidence suggests that clarity and consistency would be negatively associated with burnout, being a protective health factor, whereas constraints and consequences would be positively associated with burnout, being a risk health factor.

### 1.3. The Strong Situation Hypothesis

The strong situation hypothesis is supported by the idea that behaviour can be explained by the joint effect of personality and the characteristics of the situation [32,33]. Thus, variation in situational characteristics at work may have important effects on organisational behaviour. The degree to which the characteristics of a situation limit the effect of personality on behaviour is the degree of situational strength. The strong situation hypothesis states that a strong situation limits the effect of personality on behaviour and, as a consequence, the variability of the criterion variable is low. However, when the situation is weak, personality effect is high and also the variability of the criterion variable [34,35]. According to Meyer et al. [1,2], when the levels of the four factors (clarity, consistency, constraints, and consequences) are high, the situation is strong, prompting the individual to perform specific behaviours, which in turn will be more predictable. Conversely, when the levels of the factors are low, the situation is weak, and behaviours will be less predictable.

Some meta-analyses have provided evidence to support this hypothesis. For example, Meyer, Dalal, and Bonaccio [5] found that the personality trait of consciousness predicted both task performance and overall job performance more strongly in occupations low in constraints and consequences than in occupations high in constraints and consequences. Bowling et al. [3] achieved similar results and found that the relationship between job satisfaction and job performance was associated with constraints but not with consequences. Judge and Zapata’s results [36] revealed that all five traits were more predictive of performance for jobs in which the process by which the work was done represented weak situations. However, other authors found no support for the strong situation hypothesis. For example, Lozano [37] investigated the extent to which situational strength determines the trait level at which situations are more discriminative, finding that although situational strength moderated the effect of trait on behaviour, the results did not consistently support the situational strength hypothesis.

Although it seems to be clear that a strong situation affects behaviour, it is not so clear that it affects burnout, that is, whether a strong situation is related to less variability in burnout and whether a weak situation is related to higher variability in burnout. A strong situation implies that the levels of the four factors of situational strength are high. High values in the four variables at the same time will limit the subject to act. However, high values in the four variables at the same time will not increase or reduce burnout because high clarity and consistency will reduce burnout whereas high constraints and consequences will increase burnout. Therefore, high values in the four variables will annul or balance each other with respect to burnout.

Two issues emerge from this rationale. First, variability in the health outcome (burnout) may be not related to the strength of a situation. Second, interaction effects (moderation) between factors of situational strength are expected concerning burnout. Specifically, it could be expected that clarity and consistency effects on burnout strengthen when interacting with each other, and the effects of constraints and consequences on burnout also increase when interacting with each other. Additionally, it could be expected that constraints restrict the effect of clarity and consistency on burnout. Accordingly, we present the next hypothesis:

**Hypothesis** **3.** *Contrary to the strong situation hypothesis, a strong situation (high clarity, consistency, constraints, and consequences) will not be related to lower variability of burnout and a weak situation will not be related to greater variability of burnout.*

Additionally, we expected and tested the interaction effects between situational strength factors to explain burnout, but due to the lack of previous literature, we did not formulate any specific hypothesis.

## 2. Materials and Method

### 2.1. Participants

Three samples were collected from Ecuador. Sample 1 was made up of 136 salespeople from the pharmaceutical (61%) and information technology (39%) sector. Of these, 54.8% were men. The mean age was 36.92 years (SD = 8.28; range from 22 to 62 years) and the mean experience was 3.98 years (SD = 3.15; range from 1 to 16 years). Regarding the academic level, 34.8% had completed high school studies, 23.7% had vocational training studies, 41.5% had completed undergraduate or higher studies (master’s degree).

Sample 2 was made up of 203 office employees from different occupations, including administrative, financial, architects, and health personnel, among others. Of these, 43% were men. The mean age was 37.84 years (SD = 8.63; range from 20 to 60 years). The average experience in the current position was 7.99 years (SD = 7.09; range from 1 month to 38 years). Regarding the academic level, 33.4% completed high school studies, 18.2% had vocational training studies, and 48.3% completed undergraduate or higher studies (master’s).

Sample 3 included 168 teachers from primary (44%) and secondary (66%) education. Of these, 46.4% were men. The mean age was 40.82 years (SD = 10.37; range from 21 to 65 years). The average experience was 9.18 years (SD = 8.45; range from 1 month to 36 years). Regarding the academic level, 10.1% completed high school studies, 17.8% had vocational training studies, and 72.1% completed undergraduate or higher studies (master’s).

### 2.2. Procedure

Sample 1 (salespeople) and sample 2 (office employees) were selected through an incidental method considering sales representatives and office employees from companies in the city of Guayaquil (Ecuador). Managers from different companies were requested permission to collect data from the employees. Voluntary participation in the study and confidentially of collected data was guaranteed. Participants were given clear instructions to reliably complete the questionnaire. Paper and pencil questionnaires were scheduled to be collected without affecting working hours.

For sample 3 (teachers), we invited 20 educational institutions from the city of Guayaquil, and six of them (two public institutions and four private institutions) agreed to participate in the study. The participation of teachers was voluntary. The questionnaire was applied individually and self-administered, not affecting the teachers’ working hours. Before completing the questionnaire, participants were informed about the study objectives, they were given the instructions to adequately complete the questionnaire, and confidentiality and ethical treatment of the data were guaranteed.

### 2.3. Instruments

**Burnout**. To assess burnout, the Spanish translation [38] of the “Maslach Burnout Inventory General Survey” MBI-GS [39] was used. A single measure of burnout was computed that included five items related to emotional exhaustion (e.g., “Because of my work I feel emotionally exhausted”) and five items related to cynicism (e.g., “I think I have become more cynical in my work”). These dimensions are considered the most important manifestations of the syndrome [40] and the more reliable ones [41]. In this study, reliabilities were adequate for the three samples (see reliability values in Table 1). The responses were measured with a frequency scale ranging from 0 = “Never” to 6 = “Every day.”

**Situational strength**. We used the Situational Strength at Work Scale [2]. Specifically, clarity was measured with five items (e.g., “Specific information is given about job-related responsibilities”), consistency with five items (e.g., “All requirements are compatible with each other”), constraints with five items (e.g., “Employees are prevented from making their own decisions”), and consequences with five items (e.g., “Serious consequences occur when an employee makes a mistake”). Responses were evaluated on a six-level scale ranging from 1 = “Strongly disagree” to 6 = “Strongly agree.” The reliabilities for each variable were adequate in the three samples ranging from α = 0.69 to α = 0.95 (see Table 1). The factor structure of the questionnaire was validated. The confirmatory factor analysis of the scale had a good fit for the four-factor structure (*χ^2^* = 570, *df* = 164, *p* < 0.001, Confirmatory Fit Index CFI = 0.92, Tucker-Lewis Index TLI = 0.91, Root Mean Square Error of Approximation RMSEA = 0.06). Given that being a strong or weak situation is associated with the occupation (see for example, Bowling et al., 2015; Meyer et al., 2009) [3,5], we carried out the structural invariance analysis of the scale according to occupation. The fit values were estimated for the subsample of salespeople (*χ^2^*/*df* = 2.35, CFI = 0.887, RMSEA = 0.099), teachers (*χ^2^/df* = 2.51, CFI = 0.875, RMSEA = 0.094), and office employees (*χ^2^/df* = 2.37, CFI = 0.885, RMSEA = 0.082), finding no significant variations in the differences of *χ^2^* or in the differences of CFI between the three groups, which suggests that the scale does not vary between the three occupations.

### 2.4. Data Analysis

Separately analyses were carried out for each sample. Descriptive statistics (mean, standard deviation, and zero-order correlation) of the study variables were calculated. We performed regression analysis introducing the variables of situational strength in the first step, to test the direct effect on burnout (H1); in the second step, we introduced the two-way interactions between the factors of the situational strength; and in the third step, we introduced the three-way interactions. For the risk factor analysis (H2), the variables were dichotomised into high and low by classifying the subjects into two clusters with the maximum-likelihood distance method and in the absence of a reference cut-off point we used the mean as suggested in previous research (see for example, García-Arroyo and Osca [9]). Then, the *χ^2^* (chi-square) value and the odds ratio for each factor (crude and adjusted by sex and age) were calculated. Finally, to test the hypothesis of the strong situation (H3), we tested whether the variation of the criterion variable (burnout) in strong situations was significantly lower than in weak situations. Consequently, to compare variability of burnout between strong and weak situations we computed a new variable (Situation: strong, weak) from the high (above the mean) and low (below the mean) values of clarity, consistency, constraints, and consequences. The situation was categorised as “strong” if at least three factors of the situational strength scored high (that is, above the mean). Otherwise, the situation was categorised as “weak.” Afterward, the tests of equality of means (*t*-test) and homogeneity of the variances (Levene’s test) were carried out for the criterion variable (burnout) according to the groups: strong situation and weak situation.

## 3. Results

### 3.1. Descriptives and Correlations

Table 1 shows the descriptive statistics (mean and standard deviation) and the zero-order correlations between the study variables for the three samples.

It should be noted that in the three samples, clarity and consistency negatively correlated to burnout, whereas constraints positively correlated to burnout. These results supported hypothesis 1. Consequences did not correlate significantly with burnout in any sample. Clarity and consistency strongly and positively correlated with each other in the three samples, suggesting that they measure very similar aspects of situational strength. Regarding demographic variables, sex and age significantly correlated with burnout only in the salespeople sample.

### 3.2. Risk Factors Analysis (H2)

Table 2 shows the results of risk analyses for each factor. Clarity was found to be a protective factor in the three samples, and consistency was a protective factor only in the sample of salespeople. The constraints turned out to be a risk factor in the sample of office employees and the teachers, and consequences were not significant either as a risk factor or as a protective factor. The odds ratio estimates adjusted for sex and age did not show significant differences to the crude odds ratio values for any factors analysed. These results partially supported hypothesis 2, with differences between samples (occupations).

### 3.3. Strength and Weak Situation Analysis (H3)

To test hypothesis 3, we performed an analysis of mean differences (*t*-test) and differences in variability (Levenes’ test) between the strong situation and weak situation groups for burnout. The results showed (see Table 3) that the mean of burnout is significantly higher in the weak situation in the sample of salespeople and office employees but not in the teachers’ sample. Regarding whether the variability of burnout is lower in strong situations and higher in weak ones (strong situation hypothesis), the results showed that the standard deviation (variability) is significantly higher in the weak situation than in the strong situation but only for salespeople. No significant differences were found between the standard deviations of strong and weak situation groups for office employees and teachers. This supports hypothesis 3 for the office employees and the teachers samples.

### 3.4. Interaction Effects

To test the effect of interaction, linear regressions were performed (see results in Table 4). In the first step (model 1), we introduced the variables of the situational strength to test direct effects (H1). To test the interaction effects, in the second step, we introduced the double interactions between the four variables of the situational strength (six interactions, model 2), and in the third step (model 3) we introduced the triple interaction between clarity × consistency × constraints. We selected these variables because clarity and consistency correlated negatively to burnout, whereas constraints correlated positively, in the three samples. In this step, we tested the modulating effect of constraints concerning clarity and consistency. Demographic variables, sex and age, were not included in the regression as control variables, since they did not correlate with any of the study variables (except with burnout in salespeople, see Table 1). The predictor variables were previously grand mean centred to avoid multicollinearity difficulties [42].

The results on the direct effects are consistent with those obtained in the correlation analysis, providing additional evidence to support hypothesis 1. It should be noted that the variable that most influences in salespeople is consistency (*β* = −0.33, *p* = 0.015), clarity in office workers (*β* = −0.41, *p* = 0.001), and constrictions in teachers (*β* = 0.30, *p* = 0.001), suggesting that each occupation has a specific pattern of situational strength.

**Figure 1:**
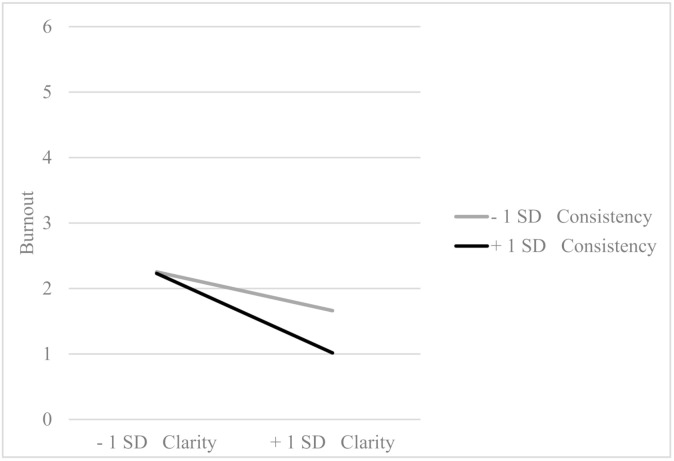
Interaction effect between clarity and consistency in the office employees’ sample (*β* = −0.16, *p* = 0.005).

About interaction effects, the results vary according to the sample. In the sample of office employees, clarity and consistency interact significantly (*β* = −0.16, *p* = 0.005) so that high consistency enhances the reducing effect of high clarity on burnout. On the other hand, if the consistency is low, the effect of clarity on burnout is significantly smaller (see Figure 1).

**Figure 2:**
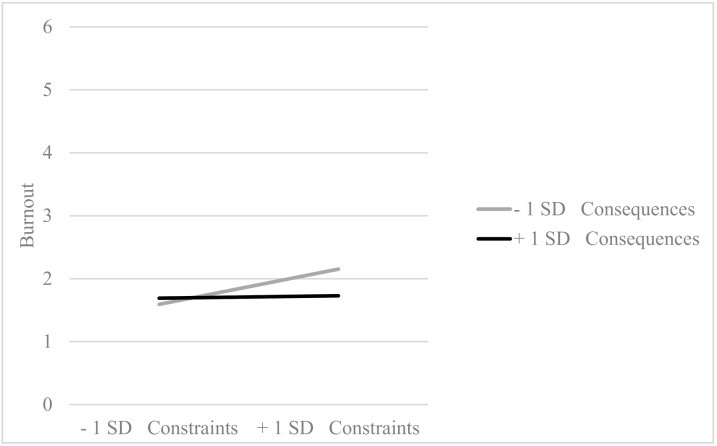
Interaction effect between constraints and consequences in the office employees’ sample (*β* = −0.11, *p* = 0.030).

**Figure 3:**
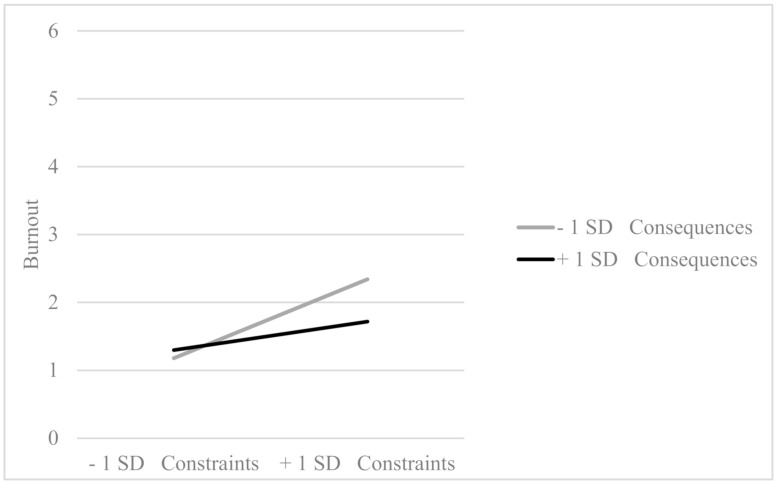
Interaction effect between constraints and consequences in the teachers’ sample (*β* = −0.14, *p* = 0.011).

Additionally, consequences modulated the effect of constraints on burnout (*β* = −0.11, *p* = 0.030), in such a way that the positive effect of high constrains on burnout is significantly attenuated when the consequences are high (Figure 2). This interaction explained a significant amount of variance of burnout (Δ*R^2^* = 0.06, *p* = 0.021). In the sample of teachers, consequences modulated the effect of constraints on burnout (*β* = −0.14, *p* = 0.011), producing the same effect that was explained for the sample of office workers (see Figure 3). This interaction explains a significant 5% of the burnout variance in teachers.

**Figure 4:**
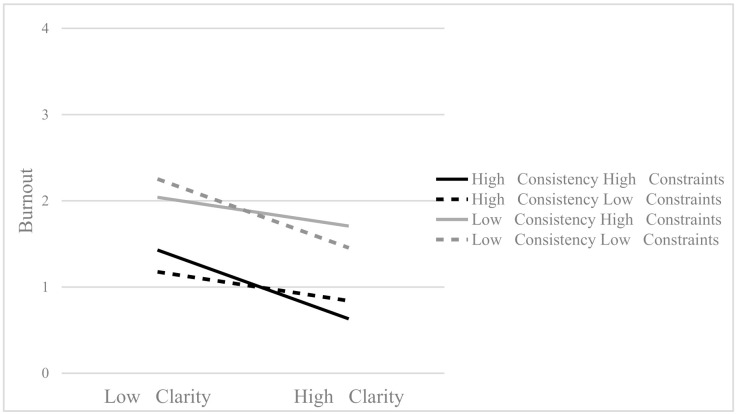
Triple interaction effect to explain burnout in the salespeople sample (*β* = −0.08, *p* < 0.05).

Finally, in the sample of salespeople, the three-way interaction between clarity, consistency, and constraints was significant (*β* = −0.08, *p* = 0.043), increasing a significant 3% the explained variance of burnout. Figure 4 shows the three-way interaction effect. Regardless of the level of consistency and constrains, burnout tends to decrease as clarity increases. However, the variation in burnout is higher when both consistency and constraints are high or when both consistency and constraints are low than when consistency is high and constraints are low or when consistency is low and constraints are high. This triple interaction indicates the weight that clarity, consistency, and constraints have for salespeople and albeit consistency has an important direct influence, clarity and constraints must also be considered for their interaction effects.

## 4. Discussion

This study aimed to analyse the relationship between situational strength and burnout. Specifically, we examined whether situational strength characteristics may be associated with burnout, whether these characteristics are risk (or protective) factors for burnout, and whether a strong situation is related to higher levels of burnout.

The results of the analyses have presented some important findings. First, clarity and consistency are negatively related to burnout while constraints are positively related to burnout in the three samples considered (H1), so that situational strength can explain between 20% and 33% of the variance of burnout. Second, these associations support that clarity and consistency are protective factors against burnout, while constraints are risk factors for burnout (H2). Third, a strong situation, comprising high clarity, consistency, constraints, and consequences, is not associated with low variability in burnout, just as a weak situation is not associated with high variability in burnout, except in the salespeople’s sample (H3). Fourth, the situational strength dimensions interact with each other, either by moderating their effects on burnout (for example, clarity with consistency, and constraints with consequences) or by adjusting the effect of one on the other (for example, the constraints balance the effect of clarity and consistency on burnout).

### 4.1. Theoretical Implications

Our findings have some important theoretical implications that we outline below. First, situational strength is not only related to behaviour but also to health. The well-known and documented idea that behaviour is a function of the interaction between the person and the environment and that, in the triad composed by person–situation–behaviour, each factor cannot be understood except by the effect of the others two [43] is expanded with the idea that the situation also has health effects, specifically on burnout. Our results provide evidence about clarity and consistency as protective factors of burnout and constraints as a risk factor of burnout. Furthermore, rewording Funder [43], one could even advanced the idea that health together with the person and the environment form a triad where each factor cannot be explained independently of the other two.

Second, the effects of situational strength on burnout are diverse depending on the situational strength factor. That is, unlike what happens with behaviour where all factors contribute to the same direction limiting action, the effects of situational strength to burnout are diverse. Thus, some facets (clarity and consistency) are negatively associated with burnout, whereas others (constraints) are positively associated with burnout. Likewise, the strength of the situation influences more or less the variability of behaviour depending on the degree of strength of a situation (strong situation hypothesis) [3,37], but this is not the case with burnout. Furthermore, some dimensions modulate the effect of the others either by enhancing (for example, consistency on clarity) or by reducing (for example, the consequences on restrictions) their effect on burnout. Even more, some dimensions can cancel out or balance the effect of others on burnout, for example, constraints balance the effect of clarity and consistency.

Third, the level of the association between situational strength and burnout can vary by occupation, as our results have shown. This is reasonable since the situations are different depending on the occupation, the tasks carried out in each occupation, the climate and organisational culture, and the temporal fluctuations [1]. Notably, the most variance of burnout is explained in the salespeople sample, specifically by clarity and consistency due to the need for clear tasks and objectives that a sale requires; however, it is striking that constraints and consequences do not influence salespeople since their work does have very clear consequences and restrictions. This would explain why, for example, constraints are a risk factor in teachers, an occupation where creativity and autonomy are essential for the teaching–learning processes [44], while they are not a risk factor for salespeople who tend to have planned sales activities and where there is little autonomy and control [45].

Fourth, the strong situation hypothesis is not conclusive. According to the theory [34,35], the variability of a criterion variable depends on the effect of interaction between personal variables and the situation, so that a strong situation can minimise the effect of the personality resulting in little variation in the criterion variable, while a weak situation would not cancel the personality effect, and as a consequence, the variation in the criterion variable would be greater. Some authors found evidence to support this hypothesis [3,36] although others did not [37]. Recently, the meta-analysis by Keeler et al. [46] found that for the most part, the differences in variance between weak and strong situations are minimal and that when differences exist, they are often in the opposite direction than expected (that is, a greater variation in “strong” situations). In line with these results, our findings showed that the variance of burnout was not significantly higher in weak situations, or lower in strong situations, except in the salespeople’s sample. The difference in burnout’s variability between strong and weak situations in the salespeople sample and the other two samples may be due to the difference of the tasks and the leadership styles of each occupation, as already indicated above. For example, the uncertainty associated with a weak situation may impact harder on the burnout of salespeople than on the burnout of the other two occupations. Further, since all three occupations involve working with other people, the differences between dealing with a client, an office user, or a student may lead to a different burnout level according to the strength of the situation. For example, the consequences of a weak situation may be risker for burnout when dealing with a client than when teaching a student. It could also occur that the vocational preferences to choose a specific profession (for example choosing to be a teacher instead of being a salesperson) and the gained experience in each occupation [47] can influence the level of impact that a strong or weak situation may have on the burnout of the subjects of each profession. Finally, it could be possible that sex and age may play a role in salespeople’s burnout results since this sample is the only one where sex and age correlate with burnout.

### 4.2. Practical Implications

Some practical implications arise from the fact that situational strength is associated with health. First, the characteristics of the situational strength can be modified by the organisation. Since organisational communication is related to job satisfaction and performance [48], informational content can be improved by increasing its clarity and improving consistency in the transmission of information to increase satisfaction and performance but also as a measure to reduce burnout. In turn, constraints can be used as management measures to improve the performance of workers [49] without realising that they can also cause burnout problems that will affect performance in the long term. Therefore, managers and Human Resource practitioners must consider health effects, in addition to performance-related ones, when environmental conditions such as situational strength characteristics are changed. Second, since situational strength factors affect burnout differently depending on the occupation, it will be important to take into account the environmental and task characteristics when designing or reorganising jobs and when planning intervention programmes on health. Third, from the interactionist point of view, situational strength has been considered as a modulator between the person and the behaviour. As we have already indicated, our results suggest that situational strength can be considered as a modulator between the person and health (burnout) and that even situational strength factors can interact with each other, modifying the direct effects that these factors may have. The ability to interact with each other, in addition to third variables, must also be considered.

### 4.3. Limitations and Strengths

Some limitations of the present study can be pointed out. First, using the operationalisation suggested by Meyer et al. [2], we have analysed in our study the characteristics of situational strength. However, other operationalisations are also possible. For example, considering the strength of a situation from the description of occupations described by the Occupational Information Network (O * NET) [50], which can give a new approach to the research on the health effects of situational strength.

Second, the objective of our study has been to analyse situational strength from the perspective of occupational health, specifically we focus on burnout and understand how the characteristics of situational strength interrelate and affect burnout. We did not intend to test the modulation hypothesis [1]. However, some predictor variable, such as conflict or role ambiguity, could have been considered in the study. Thus, future research may explore the effect of modulation of situational force between predictors and criteria variables related to occupational health. In this sense, additional covariates could have also been included, in line with previous research findings that several variables were associated with burnout (e.g., educational level, marital status, parental status, personality traits, work conditions such as workload and job insecurity [10,47,51]).

Third, the analysis of risk/protective factors as well as the test of the variability hypothesis involves transforming continuous variables into dichotomous ones. Considering variables as a continuous score is appropriate for statistical methods but not for professional practice and practitioners. It is very common to transform continuous scores into dichotomous ones differentiating between high and low levels. However, much debate exists about where to establish cut-off points [9,41]. In the absence of previous research, we have chosen the mean as the cut-off point since it is the simplest model, although other criteria are also possible based on the distribution of the sample (percentiles).

Fourth, other limitation of our study is the cross-sectional design. Considering the dynamic nature of the situations, it would be very interesting to analyse in future studies the effect of situational strength over time through a longitudinal design. We would also consider the potential biases associated with the use of self-report questionnaires for data collection. The use of this type of questionnaires has been criticised; however, self-report questionnaires are widely used in behavioural research and are accepted as long as they guarantee minimum psychometric standards of reliability and validity [52,53].

In addition to the limitations, we can also highlight some strengths of the present study. First, as far as we know, this study is the first in analysing the relationship between situational strength and burnout. As already noted, the focus on behaviour has been the most common approach when studying the situational strength. This study signifies a first step in considering the healthy or unhealthy, risky or protective effects of this construct. Second, situational strength depends on each situation and each occupation. Our study has included three samples from different occupations to better estimate this diversity, which have reinforced our results and has allowed us to detect some trends regarding the effect of the different factors of situational strength on burnout. Third, this is the first study to use the situational strength scale in Ecuador with adequate internal reliability scores as well as a good fit for the four-factor structure and a satisfactory analysis of invariance with respect to the occupation. These characteristics can be considered as a first step in the adaptation and validation of the scale in Latin American samples.

## 5. Conclusions

In the same way that situational strength, understood as clarity, consistency, constraints, and consequences, is related to behaviour, our study has evidenced that it also is associated with burnout. This focus on health, which was neglected until now in research, has important theoretical and practical implications. For example, although certain strong situations can lead to more productive behaviour, they can also lead to health problems, which in the long term would also be detrimental to performance. Therefore, it will be important for organisational psychology researchers and human resource professionals to take this point into account to gain a balance between performance and health.

## Figures and Tables

**Table 1:** Descriptives (mean, standard deviation), reliabilities (in brackets on the diagonal), and correlation coefficients.

		Mean	SD	1	2	3	4	5	6	
Salespeople *N* = 136										
1	Sex	1.44	0.50							
2	Age	37.00	8.90	−0.47 **						
3	Burnout	1.44	0.98	0.28 *	−0.26 *	(0.81)				
4	Clarity	4.73	1.09	−0.12	0.10	−0.48 **	(0.95)			
5	Consistency	4.46	1.01	−0.10	0.08	−0.51 **	0.84 **	(0.89)		
6	Constraints	3.40	1.32	0.06	−0.10	0.20 *	−0.41 **	−0.33 **	(0.88)	
7	Consequences	4.33	0.89	−0.04	−0.09	−0.03	0.04	0.06	0.23 **	(0.65)
Office workers *N* = 203										
1	Sex	1.57	0.50							
2	Age	37.84	8.63	0.08						
3	Burnout	1.75	1.07	0.03	0.04	(0.80)				
4	Clarity	4.30	1.11	−0.07	−0.01	−0.30 **	(0.89)			
5	Consistency	4.10	0.95	−0.04	−0.07	−0.14 *	0.72 **	(0.84)		
6	Constraints	3.62	1.16	0.01	0.06	0.15 *	0.01	−0.03	(0.83)	
7	Consequences	4.17	1.11	−0.13	−0.07	−0.05	0.23 **	0.14 *	0.37 **	(0.73)
Teachers *N* = 168										
1	Sex	1.62	0.49							
2	Age	40.82	10.37	−0.02						
3	Burnout	1.71	1.18	−0.06	−0.11	(0.84)				
4	Clarity	4.31	1.21	−0.09	0.14	−0.29 **	(0.91)			
5	Consistency	4.14	0.97	−0.07	0.05	−0.22 **	0.71 **	(0.82)		
6	Constraints	3.24	1.26	−0.10	0.01	0.25 **	0.06	0.12	(0.86)	
7	Consequences	3.92	1.14	−0.14	0.04	−0.01	0.11	0.23 **	0.44 **	(0.75)

* *p* < 0.05, ** *p* < 0.01.

**Table 2:** Risk factor analysis: odds ratio and confidence intervals.

	Salespeople (*N* = 136)			Office Workers (*N* = 203)			Teachers (*N* = 168)		
	*χ* ^2^	OR (CI)	OR adj (CI)	*χ* ^2^	OR (CI)	OR adj (CI)	*χ* ^2^	OR (CI)	OR adj (CI)
Clarity	24.35 ***	0.14 (0.06–0.32)	0.16 (0.07–0.36)	11.80 **	0.40 (0.21–0.66)	0.37 (0.21–0.66)	7.23 **	0.34 (0.15–0.76)	0.34 (0.15–0.76)
Consistency	13.80 ***	0.25 (0.12–0.53)	0.27 (0.13–0.60)	3.85 *	0.57 (0.32–1.01)	0.58 (0.33–1.02)	1.54	0.62 (0.29–1.32)	0.62 (0.29–1.32)
Constraints	0.89	1.41 (0.69–2.87)	1.33 (0.64–2.76)	4.46 *	1.91 (1.04–3.51)	1.90 (1.04–3.49)	9.54 **	3.42 (1.53–7.66)	3.38 (1.51–7.56)
Consequences	0.84	0.71 (0.34–1.48)	0.79 (0.37–1.67)	0.04	0.95 (0.54–1.65)	0.97 (0.55–1.70)	0.01	1.00 (0.47–2.11)	0.98 (0.46–2.09)

* *p* < 0.05; ** *p* < 0.01; *** *p* < 0.001. Note: degrees of freedom for Chi^2^ = 1. OR = odds ratio; OR adj = odds ratio adjusted by sex and age.

**Table 3:** Mean differences (*t*-test) and variability (homogeneity of variances Levenes’ test) of strong/weak situations associated with burnout.

		*N*	Mean	SD	*t* (*p*-Value)	*df*	*F* (Levenes’ Test) (*p*-Value)
Salespeople							
Strong situation		77	1.20	0.82	−3.38 (*p* = 0.001)	134	5.34 (*p* = 0.022)
Weak situation		59	1.75	1.07			
Office employees							
Strong situation		74	1.53	0.97	−2.28 (*p* = 0.023)	201	2.53 (*p* = 0.113)
Weak situation		129	1.88	1.11			
Teachers							
Strong situation		49	1.64	1.26	−0.48 (*p* = 0.630)	166	0.97 (*p* = 0.326)
Weak situation		119	1.73	1.15			

**Table 4:** Regression results of situational strength characteristics to explain burnout.

	Salespeople			Office Employees			Teachers		
	Model 1	Model 2	Model 3	Model 1	Model 2	Model 3	Model 1	Model 2	Model 3
Intercept	1.44 ***	1.51 ***	1.44 ***	1.75 ***	1.94 ***	1.94	1.71 ***	1.77 ***	1.78 ***
Clarity	−0.17 *	−0.29	−0.26	−0.41 ***	−0.44 ***	−0.43 ***	−0.26 *	−0.25 *	−0.24 *
Consistency	−0.33 *	−0.27	−0.32 *	−0.20	−0.19	−0.18	−0.06	−0.09	−0.08
Constraints	0.01	−0.07	0.01	0.16 *	0.14 *	0.10	0.30 ***	0.34 ***	0.37 ***
Consequences	−0.01	0.05	0.06	−0.05	−0.08	−0.08	−0.11	−0.12	−0.11
Clarity × Consistency		0.06	0.17		−0.16 **	−0.14 *		−0.01	−0.02
Clarity × Constraints		0.11	0.04		0.06	−0.08		−0.08	−0.08
Clarity × Consequences		−0.09	−0.11		−0.03	−0.02		0.10	0.08
Consistency × Constraints		0.07	0.12		−0.02	−0.02		0.07	0.07
Consistency × Consequences		−0.13	−0.09		0.00	−0.01		0.03	0.04
Constraints × Consequences		−0.04	−0.06		−0.11 *	−0.12 *		−0.14 *	−0.14 *
Clarity × Consistency × Constraints			−0.08 *			0.04			−0.02
*R^2^*	0.27	0.30	0.33	0.13	0.19	0.20	0.17	0.22	0.23
Δ*R^2^*		0.03	0.03 *		0.06 *	0.01		0.05 *	0.01

* *p* < 0.05; ** *p* < 0.01; *** *p* < 0.001.
